# Patient-Reported Experiences in Chronic Dermatological Conditions: Validation of the Romanian PSPSQ 2.0 Within Contemporary Dermatologic Care Pathways

**DOI:** 10.3390/diagnostics16132112

**Published:** 2026-07-06

**Authors:** Nicoleta Cirstea, Delia Mirela Tit, Mirela Marioara Toma, Anamaria Lavinia Purza, Ada Radu, Gabriela S. Bungau, Ruxandra-Cristina Marin, Călin Muntean, Georgiana Iris Tit, Radu Dumitru Moleriu

**Affiliations:** 1 Doctoral School of Biomedical Sciences, Faculty of Medicine and Pharmacy, University of Oradea, 410087 Oradea, Romania; cirstea.nicoleta@student.uoradea.ro (N.C.); adaradu@uoradea.ro (A.R.); gbungau@uoradea.ro (G.S.B.); marin.ruxandracristina@student.uoradea.ro (R.-C.M.); 2 Department of Pharmacy, Faculty of Medicine and Pharmacy, University of Oradea, 410028 Oradea, Romania; purza_lavinia@uoradea.ro; 3 Department of Pharmacology, Clinical Pharmacology and Pharmacotherapy, Faculty of Medicine, “Carol Davila” University of Medicine and Pharmacy, 050474 Bucharest, Romania; 4 Department III, Functional Science, Discipline of Medical Informatics and Biostatistics, “Victor Babes” University of Medicine and Pharmacy, 300041 Timisoara, Romania; cmuntean@umft.ro (C.M.); radu.moleriu@umft.ro (R.D.M.); 5 Faculty of Psychology and Education Sciences, University Babes-Bolyai, 400001 Cluj Napoca, Romania; georgiana.tit@stud.ubbcluj.ro

**Keywords:** patient-reported experience measures (PREMs), PSPSQ 2.0, chronic dermatological conditions, pharmaceutical care, community pharmacy, psychometric validation, patient-centered care, dermatologic care pathways

## Abstract

**Background/Objectives**: Chronic dermatological conditions increasingly require complex and patient-centered therapeutic management, including biologic therapies, injectable treatments, and multidisciplinary care. In this context, patient-reported experience measures (PREMs) may provide valuable insight into the quality and effectiveness of pharmacist-delivered care. This study aims to translate, culturally adapt, and evaluate the psychometric performance of the Patient Satisfaction with Pharmacist Services Questionnaire (PSPSQ 2.0) as a patient-reported experience measure in Romanian patients with chronic dermatological conditions. **Methods**: A cross-sectional validation study was conducted in community pharmacies across Romania (*N* = 220). The questionnaire was translated using a structured forward-translation and expert review process, in accordance with ISPOR and COSMIN recommendations. Internal consistency was assessed using Cronbach’s alpha and item-level statistics. Construct validity was examined using exploratory factor analysis (EFA), confirmatory factor analysis (CFA), and bifactor modeling. Known-groups validity and floor and ceiling effects were also evaluated. **Results**: The Romanian PSPSQ 2.0 demonstrated excellent internal consistency (α = 0.978; subscales α = 0.961–0.969). EFA indicated a dominant single-factor structure, explaining 84.0% of the variance. In CFA, the original three-factor model showed excellent relative fit (CFI = 0.999, TLI = 0.999), although RMSEA indicated some model misfit (0.109). Bifactor analysis revealed a strong general satisfaction factor, with consistently high loadings (0.80–0.99), suggesting that most item variance is attributable to a global patient satisfaction construct. These findings support the use of the instrument as a global measure of patient experience within contemporary dermatologic care pathways. **Conclusions**: The Romanian version of the PSPSQ 2.0 demonstrates excellent reliability and acceptable construct validity as a PREM for assessing patient satisfaction with pharmacist services. The findings support the use of total scores as a robust indicator of patient experience, while domain-level interpretation should be approached with caution due to substantial overlap between dimensions. This instrument may support the integration of patient-reported measures into routine evaluation of pharmaceutical care.

## 1. Introduction

Chronic dermatological conditions are common long-term disorders that can substantially affect physical comfort, sleep, emotional well-being, self-image, social functioning, and daily activities. Across skin diseases, the burden is not limited to symptoms alone, because visible lesions, itching, pain, and chronic relapse can have persistent effects on health-related quality of life [1,2].

Many chronic skin conditions also require prolonged treatment and repeated follow-up, which makes sustained treatment adherence an important part of disease control. In dermatology, adherence is often challenged by a long treatment duration, inconvenient topical regimens, delayed responses, fear of adverse effects, and a mismatch between patient expectations and treatment results. These issues are especially relevant in conditions such as psoriasis, atopic dermatitis, acne, and chronic urticaria [3,4,5].

Recent advances in dermatology have expanded therapeutic options for patients with chronic skin diseases, including biologic therapies, injectable treatments, and energy-based procedures. These developments have increased the complexity of treatment pathways and the need for ongoing patient education, monitoring, and adherence support [6,7]. In addition, energy-based technologies (EBDs) are increasingly used in the management of both aesthetic and medical dermatological conditions [8,9,10,11], further emphasizing the importance of patient-centred care and effective communication throughout the treatment process. Within this evolving healthcare landscape, patient-reported experience measures may provide valuable insights into how patients perceive and evaluate the healthcare services they receive. The growing complexity of dermatological care has also reinforced the importance of multidisciplinary approaches, in which pharmacists may contribute through medication counselling, treatment support, and patient education [12,13,14].

Because patients receiving advanced dermatologic therapies often require repeated counseling, long-term monitoring, and continuous interaction with healthcare professionals, the assessment of patient-reported experiences and satisfaction has become increasingly relevant. In this context, validated patient-reported outcome and experience measures may provide valuable information regarding the quality and patient-centeredness of pharmacist services within modern dermatologic care models [15].

In this context, community pharmacists are well positioned to support patients with chronic dermatological diseases. Pharmacists are often the most accessible healthcare professionals and may contribute through counseling on correct medicine use, reinforcement of adherence, management of adverse effects, support for self-care, and identification of treatment-related problems that require referral. Recent dermatology-focused pharmacy literature has also highlighted the contribution of pharmacists to medication access, education, and monitoring in skin disease management [14,16].

More broadly, pharmacy practice has increasingly moved from a product-centered model toward a patient-centered model that emphasizes clinical services, communication, and longitudinal support. Pharmacist-led interventions in ambulatory care have been associated with improvements in patient-related outcomes, including behavioral and humanistic outcomes, which supports the need to evaluate pharmacy services not only by technical quality but also by patient experience [17].

Patient satisfaction is an important patient-reported outcome and a recognized indicator of healthcare quality. In pharmacy settings, satisfaction reflects how patients perceive communication, trust, accessibility, responsiveness, and the usefulness of counseling. It is also relevant because patient perceptions of pharmacy services may influence confidence in treatment, service use, and adherence-related behaviors [18,19]. In dermatology, patient-reported outcomes (PROs) and patient-reported experience measures are increasingly recognized as important complementary tools for evaluating outcomes associated with advanced therapeutic and procedural interventions, including biologic therapies and energy-based treatments [20].

Also, patient satisfaction is part of the broader category of PROs, which capture patients’ perspectives on health status, treatment, and healthcare experiences without external interpretation. PRO measures are increasingly recommended in healthcare evaluation because they provide complementary information to clinical outcomes and support patient-centered decision-making [21]. The assessment of satisfaction with pharmacist services therefore requires instruments with adequate reliability and validity. The Patient Satisfaction with Pharmacist Services Questionnaire (PSPSQ 2.0), developed by Sakharkar et al., is one of the best-known instruments designed for this purpose. It assesses three domains: Quality of Care, Interpersonal Relationship, and Overall Satisfaction, and the original study reported good psychometric performance across clinical pharmacy settings [22]. Using standardized and validated questionnaires is important because measurement instruments should show adequate measurement properties, including validity and reliability, before they are used to support research conclusions or service evaluation. This is especially important when an instrument is applied in a population or language different from the one in which it was originally developed [23,24].

The PSPSQ 2.0 has continued to be used and adapted in different healthcare and linguistic contexts. Recent work has supported the feasibility of culturally adapted versions, including an Arabic version and a shortened Traditional Chinese version, showing that the core construct remains useful across settings, although local validation is necessary before use in a new population [25,26].

Cross-cultural adaptation of patient-reported outcome measures is not a simple linguistic exercise. It requires attention to semantic, conceptual, and contextual equivalence so that the translated instrument measures the same construct in the target population. For this reason, structured methodological frameworks are recommended. The ISPOR principles of good practice remain a foundational reference for translation and cultural adaptation, while the COSMIN reporting guideline provides more recent standards for studies evaluating the measurement properties of patient-reported outcome measures [27,28].

According to COSMIN, content validity is the most important measurement property of a patient-reported outcome measure, and psychometric evaluation after translation is needed to examine whether the adapted version still performs adequately in the target context. In practice, this means that structural validity, internal consistency, and other relevant measurement properties should be examined rather than assuming that linguistic translation alone is sufficient [29,30].

In Romania, interest in patient satisfaction with community pharmacy services has increased, and recent studies have examined patient perceptions of pharmacy care in Romanian settings. However, these studies used local questionnaires or service-specific measures rather than a formally translated and psychometrically validated Romanian version of the PSPSQ 2.0. To our knowledge, no validated Romanian PSPSQ 2.0 is currently available for use in research or practice [31,32]. This gap is important in patients with chronic dermatological conditions, because they often need repeated medicine use, ongoing counseling, and practical support with treatment routines. Measuring their satisfaction with pharmacist services may help identify strengths and limitations in community pharmacy care and may support service improvement in a patient-centered direction [1,14].

Therefore, our research considered to translate, culturally adapt, and psychometrically validate the PSPSQ 2.0 for use in Romanian patients with chronic dermatological conditions. In addition to linguistic adaptation, the study sought to evaluate the reliability, construct validity, and structural characteristics of the instrument within this specific patient population. By providing a validated Romanian version of the PSPSQ 2.0, this research aims to facilitate the systematic assessment of patient satisfaction with pharmacist services and to support the development and evaluation of patient-centered pharmacy care in both clinical practice and future research settings.

## 2. Materials and Methods

### 2.1. Study Design and Participants

A cross-sectional psychometric validation study was conducted to translate, culturally adapt, and evaluate the measurement properties of the PSPSQ 2.0 in a Romanian-speaking population. The study was designed in accordance with COSMIN recommendations for patient-reported outcome measures [33] and is reported following the STROBE guidelines [34]. Participants were recruited consecutively from five community pharmacies belonging to national pharmacy chains located in Oradea, Romania. These pharmacies were selected based on their proximity to hospitals and dermatology outpatient clinics and their willingness to participate in the study. Recruitment was conducted between January 2025 and January 2026. The questionnaire was administered in paper format and completed by participants on site after receiving study information and providing written informed consent.

Eligible participants were adults (≥18 years) with a physician-diagnosed chronic dermatological condition who were currently receiving dermatological treatment and had received pharmacist consultation. For the purposes of this study, chronic dermatological conditions were defined as physician-diagnosed skin diseases requiring ongoing management through pharmacological treatment and/or regular medical follow-up. A total of 300 questionnaires were distributed, of which 233 were returned. After excluding incomplete responses, 220 questionnaires were included in the final analysis. This sample size exceeded both the minimum recommended by the developers (≥100) [22] and the commonly used subject-to-item ratio of at least 10:1 for factor analysis [35].

Ethical approval was obtained from the Research Ethics Committee of the Faculty of Medicine and Pharmacy, University of Oradea, Romania (Approval No. CEFMF/4/31.05.2024), and all participants provided informed consent prior to participation.

### 2.2. Instrument and Cross-Cultural Adaptation

The PSPSQ 2.0 was used with permission from the original developers [22]. The instrument consists of 19 items and one global rating item, covering three conceptual domains: Quality of Care, Interpersonal Relationship, and Overall Satisfaction. Responses are recorded on a 4-point Likert scale ranging from “strongly disagree” (1) to “strongly agree” (4), with higher scores indicating greater satisfaction. Sociodemographic and clinical characteristics were collected alongside the questionnaire.

**Figure 1:**
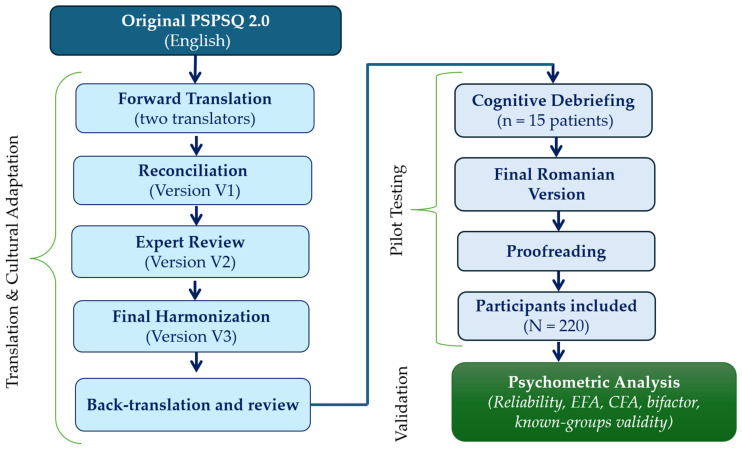
Overview of the translation, cultural adaptation, and validation process.

The translation and cultural adaptation followed a structured multi-step process based on ISPOR guidelines [27]. Two independent forward translations were produced and reconciled into a single version, followed by expert review and final harmonization. The pre-final version was pilot-tested through cognitive debriefing with 15 patients from the target population, and minor revisions were made before finalization. An additional back-translation step was performed by an independent bilingual translator blinded to the original version. The back-translated version was compared with the original questionnaire to ensure conceptual equivalence, and no major discrepancies were identified. A detailed overview of the translation and adaptation process is provided in Table 1, while the overall study workflow is illustrated in Figure 1. This approach is consistent with alternative cross-cultural adaptation methodologies emphasizing iterative forward translation and expert consensus [36].

The Romanian version of the PSPSQ 2.0 is provided in the Supplementary Materials (File S1).

### 2.3. Statistical Analysis

Statistical analyses were performed using RStudio (version 4.3.2) and JASP (version 0.19.3). Internal consistency was assessed using Cronbach’s alpha, corrected item–total correlations, and mean inter-item correlations.

Construct validity was evaluated using both exploratory factor analysis (EFA) and confirmatory factor analysis (CFA). EFA was conducted using principal axis factoring with oblimin rotation, and parallel analysis was used to determine the number of factors to retain. Factor loadings ≥0.40 were considered meaningful.

CFA was performed using diagonally weighted least squares (DWLS) to test the original three-factor structure and a unidimensional model. Model fit was evaluated using the Comparative Fit Index (CFI) and Tucker–Lewis Index (TLI), with values ≥ 0.90 considered acceptable, as well as the Root Mean Square Error of Approximation (RMSEA) and Standardized Root Mean Square Residual (SRMR), based on established thresholds.

To further investigate the dimensionality of the instrument, a bifactor confirmatory factor analysis was also performed. The bifactor model specified a general satisfaction factor loading on all items together with orthogonal domain-specific factors corresponding to the original PSPSQ 2.0 domains. Model fit was evaluated using the same indices as the CFA models (CFI, TLI, RMSEA, and SRMR). Standardized factor loadings, factor variances, and residual variances were examined to assess the relative contribution of the general and domain-specific factors and to identify potential estimation problems, such as unstable parameter estimates or Heywood cases.

Known-groups validity was assessed by comparing satisfaction scores across sociodemographic subgroups using independent samples *t*-tests and one-way ANOVA, with effect sizes calculated using Cohen’s d and eta squared (η^2^). Floor and ceiling effects were also examined, with values exceeding 15% considered indicative of limited discriminative ability.

## 3. Results

### 3.1. Participant Characteristics

A total of 233 patients participated in the study. After excluding incomplete questionnaires, the final sample included 220 participants. The sample was predominantly female (66.4%) and from urban areas (65.5%), with most participants aged between 18 and 49 years. The most frequently reported dermatological conditions were eczema (31.4%), acne (24.5%), and psoriasis (23.2%), as shown in Table 2.

### 3.2. Internal Consistency

The Romanian PSPSQ 2.0 demonstrated excellent internal consistency according to Table 3. Cronbach’s alpha was 0.978 for the total scale, with similarly high values across subscales (0.961–0.969). Corrected item–total correlations ranged from 0.802 to 0.864. Mean inter-item correlations were high, particularly within the Overall Satisfaction domain (0.900), suggesting a high degree of similarity between items. Removal of individual items did not meaningfully improve reliability.

### 3.3. Reliability, Convergent and Discriminant Validity

Convergent and discriminant validity of PSPSQ 2.0 are presented in Table 4. Convergent validity was supported by high average variance extracted (AVE) values across all three domains (0.761–0.901). However, inter-factor correlations were high (r = 0.778–0.883), and heterotrait–monotrait ratio (HTMT) values approached or exceeded recommended thresholds (0.788–0.884), indicating substantial overlap between domains and only partial discriminant validity.

### 3.4. Exploratory Factor Analysis (EFA)

Exploratory factor analysis was conducted using principal axis factoring with oblimin rotation and parallel analysis. The results indicated a dominant single-factor structure. The first factor had a substantially higher eigenvalue (16.07) and explained 84.0% of the total variance, whereas the second factor had an eigenvalue below 1 (0.95) and accounted for only 4.4% of the variance, indicating the absence of a meaningful second dimension. All items loaded strongly on the first factor, with loadings ranging from 0.87 to 0.96 (Table 5).

**Figure 2:**
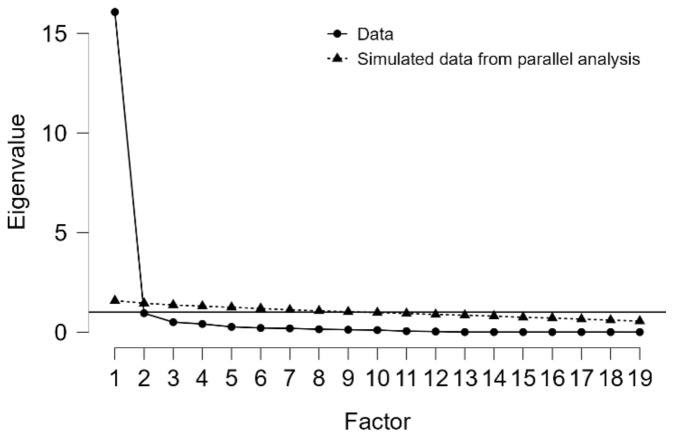
Scree plot with parallel analysis results for the PSPSQ 2.0.

These findings suggest that the PSPSQ 2.0 operates as a largely unidimensional measure in this population. The scree plot displays the observed eigenvalues (solid line) and those obtained from parallel analysis (dashed line). Only the first factor exceeded the corresponding simulated eigenvalues, supporting a unidimensional structure of the instrument (Figure 2).

### 3.5. Confirmatory Factor Analysis

The three-factor model demonstrated excellent relative fit (CFI = 0.999, TLI = 0.999) and acceptable residual fit (SRMR = 0.041), although RMSEA remained elevated (0.109), indicating some degree of model misfit. The chi-square test was significant (χ^2^(149) = 535.04, *p* < 0.001), indicating some discrepancy between the model and the observed data. Standardized factor loadings were high across all items, supporting strong convergent validity. Full standardized factor loadings for the three-factor model are presented in Table S1 (Supplementary Materials). However, inter-factor correlations were high, suggesting substantial overlap between domains.

A unidimensional model demonstrated poorer fit (CFI = 0.741, TLI = 0.709, RMSEA = 0.213, SRMR = 0.072), confirming that a simple single-factor structure does not adequately capture the covariance structure of the data.

To further investigate dimensionality, a bifactor model was tested. This model demonstrated excellent global fit (CFI = 0.999, TLI = 0.999, RMSEA = 0.099, SRMR = 0.037). Standardized loadings on the general factor were consistently high (0.80–0.99), indicating that the majority of variance in item responses is explained by a single underlying construct of patient satisfaction. In contrast, loadings on specific factors were substantially smaller, indicating that most item variance is attributable to the general factor (Table 6).

In contrast, loadings on domain-specific factors were substantially lower and less stable. The interpersonal relationship factor showed weak loadings and non-significant variance, and one item exhibited a negative loading. Additionally, the presence of Heywood cases suggests estimation instability in the specific factors.

These findings indicate that, although the theoretical three-domain structure is retained, the PSPSQ 2.0 operates largely as a unidimensional measure in this population, with limited additional contribution from domain-specific factors. A comparison of model fit indices across the tested structures is presented in Table 7.

### 3.6. Known-Groups Validity

The instrument demonstrated known-groups validity. Participants from urban areas reported significantly higher satisfaction compared to those from rural areas (64.33 ± 8.70 vs. 60.25 ± 10.57, *p* = 0.002, d = 0.435).

Satisfaction scores also differed significantly across education levels (*p* = 0.003, η^2^ = 0.064), with higher scores observed among participants with postgraduate education. Post hoc analysis indicated significantly higher satisfaction among participants with postgraduate education compared to those with high school education (*p* = 0.020) (Table 8).

### 3.7. Floor and Ceiling Effects and Feasibility

Floor effects were negligible across all domains. In contrast, ceiling effects were substantial, ranging from 24.5% for the total score to 40.9% for Overall Satisfaction, exceeding the recommended 15% threshold and indicating limited discrimination at higher levels of satisfaction.

All domain scores deviated significantly from normality (Shapiro–Wilk *p* < 0.001), with negatively skewed distributions reflecting clustering of responses toward higher satisfaction levels. Item-level descriptive statistics are provided in Table 9.

Most participants reported that the questionnaire was easy or very easy to complete, with an average completion time of 10–15 min, supporting its feasibility in routine practice.

## 4. Discussion

In increasingly complex dermatologic care pathways, patient-reported experience measures may provide clinically relevant information regarding communication quality, treatment support, and long-term therapeutic engagement. Within this context, the assessment of patient satisfaction with pharmacist-delivered care may contribute to the evaluation of patient-centered pharmaceutical services in chronic dermatological conditions. This study represents the first translation, cultural adaptation, and psychometric validation of the PSPSQ 2.0 in a Romanian population with chronic dermatological conditions. The findings demonstrate excellent internal consistency, acceptable but not fully optimal construct validity, and meaningful known-groups discrimination while also highlighting structural characteristics such as strong inter-factor correlations, ceiling effects, and the presence of a dominant general factor underlying the instrument. These results are broadly consistent with previous cross-cultural validation studies of patient satisfaction instruments in pharmacy practice, which report similarly high reliability and challenges in clearly separating satisfaction domains across different healthcare contexts [28].

The Romanian PSPSQ 2.0 demonstrated excellent internal consistency, with a Cronbach’s alpha of 0.978 for the total scale and similarly high values across all subscales, indicating that the items are highly correlated and consistently capture the underlying construct of patient satisfaction in this population [37]. However, very high internal consistency may also reflect item redundancy, particularly when inter-item correlations are elevated, as Cronbach’s alpha can be inflated by substantial semantic or conceptual overlap between items, potentially leading to overestimation of reliability [38]. Similarly, excessively high reliability coefficients may indicate limited discriminant capacity between items, suggesting the need for further evaluation of the scale structure and potential item reduction [39]. While high internal consistency supports score stability and the use of the total scale in applied settings [40], reliability coefficients approaching 1.0 should not be interpreted solely as evidence of superior measurement quality. In the present study, the particularly high mean inter-item correlations observed within domains further support the possibility that some items capture closely overlapping aspects of patient experience rather than clearly distinct facets, and this should be considered a potential limitation of the instrument.

Exploratory factor analysis indicated a dominant single-factor structure, with all items loading strongly on a general factor explaining the majority of variance. This finding suggests that patient satisfaction is primarily perceived as a global construct rather than clearly differentiated domains in this population [41]. Furthermore, empirical studies have shown that global satisfaction scores are highly influenced by interpersonal interactions with healthcare providers, leading to substantial overlap between dimensions and supporting the interpretation of a dominant general factor [42].

In contrast, confirmatory factor analysis indicated that the original three-factor model provided excellent relative fit according to the CFI (0.999) and TLI (0.999), together with a low residual error (SRMR = 0.041). However, the elevated RMSEA value (0.109) and the significant χ^2^ statistic (χ^2^/df = 3.59) suggest that model fit was not uniformly optimal across all indices. This apparent discrepancy warrants careful interpretation. While incremental fit indices supported the proposed factor structure, the RMSEA results indicate some degree of model misfit and suggest that the distinction between domains may be less pronounced than suggested by the proposed three-factor solution. It should also be noted that RMSEA is sensitive to sample size, model complexity, and departures from multivariate normality, particularly in instruments with highly correlated items. Therefore, the CFA findings provide support for the original conceptual structure, although the empirical separation of the three domains appears less clear-cut than implied by the theoretical model. Recent methodological evidence emphasizes that the evaluation of CFA models should be based on the overall pattern of fit indices rather than any individual index alone [43].

The discrepancy between exploratory and confirmatory findings suggests that the conceptual structure of the PSPSQ 2.0 is retained, but that the empirical distinction between domains is less pronounced. This difference can be explained by the fact that exploratory methods are driven by shared variance between items, whereas confirmatory approaches test predefined theoretical structures [44,45].

The bifactor model provided additional insight into the dimensionality of the instrument, demonstrating excellent overall fit and a strong general satisfaction factor underlying all items. Standardized loadings on the general factor were consistently high, whereas domain-specific factors contributed relatively little unique variance. The interpersonal relationship factor, in particular, showed weak and unstable loadings, including a non-significant variance estimate and a negative item loading. The presence of a negative loading (Heywood case) and unstable parameter estimates suggests that the domain-specific factors may not represent fully independent constructs in this sample. Consequently, interpretations based on individual subscale scores should be made with caution. These findings indicate that the PSPSQ 2.0 primarily captures a global perception of patient satisfaction, with domain-specific distinctions being less pronounced at the empirical level.

From a theoretical perspective, this pattern is consistent with hierarchical models of patient satisfaction, in which a dominant general factor underlies more specific domains. Taken together, the bifactor findings support the presence of a strong general satisfaction factor underlying the instrument, whereas evidence for clearly differentiated domain-specific constructs was comparatively limited. This interpretation is consistent with recent psychometric research demonstrating that bifactor models frequently reveal a dominant general factor alongside comparatively weaker domain-specific factors [46].

An additional finding that warrants consideration is the presence of substantial ceiling effects. Ceiling effects ranged from 24.5% for the total score to 40.9% for the Overall Satisfaction domain, considerably exceeding the commonly recommended 15% threshold for acceptable targeting [24]. Such findings suggest limited discrimination among respondents reporting very positive experiences and may reduce the instrument’s sensitivity to detect further improvements in satisfaction [24,47].

The observed response clustering may partly reflect genuinely high levels of satisfaction with pharmacist services in this population; however, it may also be influenced by characteristics of the response format. The PSPSQ 2.0 uses a four-point Likert scale without a neutral midpoint, which may encourage respondents to select positive response categories and contribute to score concentration at the upper end of the scale [48]. Similar ceiling effects have been reported in patient satisfaction instruments [49] and should be considered when interpreting the responsiveness and discriminative capacity of the scale. This pattern is consistent with the negatively skewed score distributions observed across domains and the reduced variability among highly satisfied respondents.

The strong correlations observed between factors further support the interpretation that the instrument captures closely related aspects of a broader satisfaction construct [50]. Similar patterns have been reported in recent validation studies, where high inter-factor correlations were interpreted as evidence that theoretically distinct domains are not clearly separable at the empirical level [51,52]. This pattern also supports the potential use of higher-order or bifactor models in future research, which may better account for both general and domain-specific variance.

The predominance of a strong general satisfaction factor observed in the present study may also reflect the increasingly integrated and patient-centered nature of contemporary dermatologic care. Chronic inflammatory and immune-mediated skin diseases are progressively managed using personalized therapeutic approaches, including biologic therapies, injectable treatments, lasers, and other EBDs, which often require continuous patient education, monitoring, and multidisciplinary support [7,53,54]. Within these complex therapeutic pathways, patients may perceive healthcare experiences more holistically, placing substantial emphasis on communication quality, accessibility, continuity of care, and confidence in healthcare professionals rather than clearly separating specific dimensions of service delivery. In this context, pharmacists may play an increasingly relevant supportive role through medication counseling, adherence reinforcement, education regarding injectable therapies, management of adverse effects, and guidance related to post-procedural care [12].

The strong overlap between satisfaction domains identified in the Romanian PSPSQ 2.0 may therefore partly reflect the interconnected nature of these patient experiences, particularly in chronic dermatological conditions requiring long-term therapeutic engagement. Moreover, the use of validated patient-reported experience measures may support quality assessment and patient-centered evaluation within evolving precision dermatology care models, where treatment complexity and patient expectations continue to increase [20,55,56].

The instrument demonstrated known-groups validity by distinguishing between patient subgroups defined by residence and education level. Effect sizes indicated small to moderate differences between groups. Higher satisfaction scores among urban participants and those with higher education may reflect differences in healthcare access, communication, and expectations. Previous research has shown that sociodemographic factors influence patient satisfaction by shaping both expectations and perceptions of care [18]. These findings support the sensitivity of the instrument to contextual and socioeconomic differences, reinforcing its potential utility for identifying disparities in patient experience across healthcare settings [57]. The observed differences should be interpreted cautiously, as unmeasured contextual factors (e.g., pharmacy workload, consultation time, or service availability) may also contribute to the variation.

At the same time, high satisfaction scores may reflect genuinely positive perceptions of pharmacist services, suggesting that ceiling effects should not be interpreted solely as a measurement limitation but also as an indicator of favorable care experiences [50]. The use of a 4-point Likert scale without a neutral midpoint may have further contributed to score clustering and reduced variability.

This study has several strengths, including a structured translation and cultural adaptation process, an adequate sample size, and the combined use of exploratory and confirmatory factor analysis. This approach is recommended in contemporary psychometric research, as it allows for a more comprehensive evaluation of measurement properties [58]. Furthermore, adherence to COSMIN and STROBE recommendations enhances the methodological rigor and transparency of the study.

Several limitations should be acknowledged. Although the three-factor model demonstrated the best relative fit, overall model fit indices suggest that further structural refinement may be warranted. The very high internal consistency coefficients and elevated inter-item correlations also suggest potential item redundancy, which may limit the discriminative contribution of individual items. Second, test–retest reliability was not assessed, limiting conclusions regarding the temporal stability of the instrument. Additionally, although convergent and discriminant validity were examined within the measurement model using AVE and HTMT indices, they were not evaluated against external validated instruments, limiting direct comparison with other measures of patient satisfaction and patient-reported experience [59]. The study was conducted in a specific patient population, which may limit generalizability; however, this may also be considered a strength, as it allows for a more precise evaluation within a defined clinical context [60]. Furthermore, the cross-sectional design precludes assessment of responsiveness to change. Although bifactor modeling provided additional insight into the dimensionality of the instrument, estimation issues were observed, suggesting that the domain-specific structure may require further refinement. These limitations are consistent with current methodological recommendations, which emphasize the need for further structural validation, assessment of temporal stability, and replication in diverse populations [61,62].

From a practical perspective, the Romanian PSPSQ 2.0 appears to be a useful tool for assessing patient satisfaction with pharmacist services in community pharmacy settings. The findings support the use of total scores as a robust overall measure of satisfaction, particularly in complex care settings where patient experience may be perceived more globally rather than as strictly separated service dimensions [18]. The instrument could be integrated into routine quality assessment frameworks to monitor patient experience and inform service improvements. Its relevance may further increase as dermatology continues to incorporate biologic therapies, injectable treatments, and energy-based procedures, which often require sustained adherence, patient education, and multidisciplinary support. In this context, validated instruments such as the Romanian PSPSQ 2.0 may provide useful patient-centered metrics for evaluating pharmacy services within increasingly specialized dermatologic care pathways.

Future research should aim to confirm these findings in more diverse populations, assess test–retest reliability, and explore potential refinements of the instrument. Shorter versions or revised response formats may help reduce redundancy and ceiling effects, while advanced psychometric approaches such as item response theory or Rasch analysis could further optimize scale performance.

## 5. Conclusions

The Romanian version of the PSPSQ 2.0 demonstrated excellent internal consistency and generally supportive evidence of structural and construct validity, although several findings, including ceiling effects, potential item redundancy, and instability in the bifactor solution, warrant cautious interpretation. While the original three-factor model showed the best relative fit, results from bifactor analysis indicate that the instrument is largely dominated by a general satisfaction factor, with limited distinct contribution from domain-specific dimensions.

These findings support the use of the total PSPSQ 2.0 score as a robust overall indicator of patient satisfaction with pharmacist services, whereas domain-specific scores should be interpreted with caution due to substantial overlap between dimensions. The instrument appears feasible for use in Romanian community pharmacy settings and may support the evaluation of patient-centered pharmaceutical care.

However, ceiling effects, potential item redundancy, and the absence of test–retest reliability assessment should be considered when interpreting results. Further research is warranted to evaluate temporal stability, examine external convergent and discriminant validity using established instruments, and explore potential structural refinements, including shorter or alternative versions of the scale and validation in more diverse patient populations and healthcare settings.

## Figures and Tables

**Table 1:** Translation and cross-cultural adaptation process of the PSPSQ 2.0.

Stage	Description
Stage 1: Forward translation	Two independent bilingual translators with healthcare expertise produced forward translations from English into Romanian. Translators were blinded to each other’s versions.
Stage 2: Reconciliation	The two forward translations were compared and reconciled into a single harmonized version (V1) through discussion and consensus within the research team.
Stage 3: Expert review	A multidisciplinary expert panel (pharmacists, dermatologists, and linguists) reviewed V1 for conceptual equivalence and clarity, resulting in version V2. The “Interpersonal Relationship” domain was adapted to “Interacțiunea cu farmacistul” (Interaction with the Pharmacist) to reflect the Romanian pharmacy context.
Stage 4: Back-translation	An independent bilingual translator, blinded to the original questionnaire, translated the Romanian version back into English. The back-translated version was compared with the original instrument to assess conceptual equivalence. No major discrepancies were identified.
Stage 5: Final expert harmonization	A senior academic pharmacist with expertise in research methodology conducted a final harmonization, producing version V3 with confirmed linguistic clarity and conceptual consistency.
Stage 6: Cognitive debriefing	Version V3 was pilot-tested with 15 patients from the target population to assess clarity, comprehension, and cultural appropriateness. No major issues were identified.
Stage 7: Finalization	Minor revisions were made based on patient feedback, and the final Romanian version of the PSPSQ 2.0 was established by consensus.
Stage 8: Proofreading	The final version was checked for spelling, grammar, and formatting prior to psychometric analysis.

**Table 2:** Participants’ characteristics (*N* = 220).

Characteristic	*n*	%
Gender		
Female	146	66.4
Male	74	33.6
Residence		
Urban	144	65.5
Rural	76	34.5
Age group (years)		
18–29	55	25.0
30–39	56	25.5
40–49	45	20.5
50–59	37	16.8
≥60	27	12.3
Education level		
Primary school	10	4.5
High school	92	41.8
University	104	47.3
Postgraduate	14	6.4
Dermatological condition		
Eczema	69	31.4
Acne	54	24.5
Psoriasis	51	23.2
Rosacea	29	13.2
Atopic dermatitis	13	5.9
Seborrheic dermatitis	2	0.9
Vitiligo	2	0.9

**Table 3:** Internal consistency reliability (*N* = 220).

Scale	Items	α	Mean IIC	CITC Range	α If Deleted
Total PSPSQ 2.0	Q1–Q19	0.978	0.710	0.802–0.864	0.976–0.978
Quality of care	Q1–Q10	0.969	0.775	0.805–0.862	0.964–0.969
Interpersonal relationship	Q11–Q16	0.961	0.805	0.802–0.847	0.950–0.961
Overall satisfaction	Q17–Q19	0.964	0.900	0.829–0.849	0.937–0.964

**Table 4:** Reliability, convergent validity, and discriminant validity of the PSPSQ 2.0.

Domain	Cronbach’s α	McDonald’s ω	AVE	QoC	IPR	OVS
QoC	0.969	0.967	0.761	1.000	-	-
IPR	0.961	0.962	0.804	0.814	1.000	-
OVS	0.964	0.965	0.901	0.788	0.884	1.000

Values below the diagonal represent HTMT ratios. AVE, average variance extracted; QoC = Quality of Care; IPR = Interpersonal Relationship; OVS = Overall Satisfaction.

**Table 5:** Summary of exploratory factor analysis loadings for the PSPSQ 2.0.

Domain	Item	Factor Loading	Uniqueness
QoC	Q1	0.918	0.128
	Q2	0.939	0.117
	Q3	0.931	0.072
	Q4	0.926	0.125
	Q5	0.889	0.174
	Q6	0.945	0.048
	Q7	0.957	0.049
	Q8	0.890	0.125
	Q9	0.916	0.135
	Q10	0.874	0.215
IPR	Q11	0.937	0.077
	Q12	0.906	0.117
	Q13	0.897	0.167
	Q14	0.878	0.154
	Q15	0.878	0.208
	Q16	0.939	0.030
OVS	Q17	0.936	0.078
	Q18	0.928	0.078
	Q19	0.918	0.112

Factor loadings are based on principal axis factoring with oblimin rotation. Only the dominant factor is presented, as the second factor had an eigenvalue < 1 and was not retained. QoC = Quality of Care; IPR = Interpersonal Relationship; OVS = Overall Satisfaction.

**Table 6:** Bifactor model standardized loadings for the PSPSQ 2.0 (*N* = 220).

Domain	Item	General Factor (G)	Specific Factor	Residual Variance
QoC	Q1	0.87	0.40	0.09
	Q2	0.91	0.33	0.07
	Q3	0.85	0.52	0.01
	Q4	0.88	0.44	0.04
	Q5	0.81	0.44	0.15
	Q6	0.86	0.47	0.03
	Q7	0.90	0.40	0.03
	Q8	0.80	0.48	0.14
	Q9	0.86	0.38	0.11
	Q10	0.82	0.33	0.22
IPR	Q11	0.99	0.12	0.00
	Q12	0.93	0.26	0.07
	Q13	0.91	0.27	0.09
	Q14	0.91	0.53	−0.10
	Q15	0.95	−0.06	0.09
	Q16	1.00	0.15	−0.02
OVS	Q17	0.97	0.23	0.01
	Q18	0.95	0.30	0.00
	Q19	0.96	0.25	0.02

Standardized loadings are reported. G = general satisfaction factor; QoC = Quality of Care; IPR = Interpersonal Relationship; OVS = Overall Satisfaction. Residual variances < 0 indicate Heywood cases.

**Table 7:** Comparison of confirmatory factor analysis models.

Model	χ^2^ (df)	CFI	TLI	RMSEA	SRMR
Three-factor	535.04 (149)	0.999	0.999	0.109	0.041
One-factor	1666.40 (152)	0.741	0.709	0.213	0.072
Bifactor	418.63 (133)	0.999	0.999	0.099	0.037

CFI = Comparative Fit Index; TLI = Tucker–Lewis Index; RMSEA = Root Mean Square Error of Approximation; SRMR = Standardized Root Mean Square Residual.

**Table 8:** Known-groups comparisons (*N* = 220).

Variable	Group	*n*	Mean ± SD	*p* Value	Effect Size
Residence	Urban	144	64.33 ± 8.70	0.002	d = 0.435
	Rural	76	60.25 ± 10.57		
Education level	Primary school	10	57.60 ± 8.30	0.003	η^2^ = 0.064
	High school	92	61.10 ± 9.15		
	University	104	64.24 ± 9.43		
	Postgraduate	14	68.93 ± 10.26		

*p* values were obtained using independent samples *t*-test (residence) and one-way ANOVA (education level). Effect sizes are reported as Cohen’s d and eta squared (η^2^).

**Table 9:** Item-level descriptive statistics for the Romanian PSPSQ 2.0.

Domain.	Item	Mean	SD	Skewness	Kurtosis	Ceiling (%)
QoC	Q1	3.291	0.579	−0.557	1.826	34.1
	Q2	3.300	0.566	−0.384	1.181	34.5
	Q3	3.314	0.571	−0.568	1.976	35.5
	Q4	3.295	0.588	−0.585	1.667	35.0
	Q5	3.232	0.631	−0.557	0.939	32.7
	Q6	3.291	0.563	−0.361	1.234	33.6
	Q7	3.318	0.548	−0.331	1.380	35.0
	Q8	3.209	0.703	−0.556	0.030	35.9
	Q9	3.195	0.671	−0.434	−0.024	33.2
	Q10	3.055	0.792	−0.543	−0.126	30.5
IPR	Q11	3.377	0.539	−0.026	−0.960	40.5
	Q12	3.395	0.560	−0.210	−0.847	43.2
	Q13	3.368	0.537	0.007	−0.942	39.5
	Q14	3.395	0.543	−0.093	−0.985	42.3
	Q15	3.295	0.588	−0.449	0.919	35.5
	Q16	3.336	0.570	−0.451	1.112	37.7
OVS	Q17	3.423	0.539	−0.129	−1.093	44.5
	Q18	3.418	0.571	−0.489	0.187	45.5
	Q19	3.414	0.579	−0.656	1.096	45.0

QoC = Quality of Care; IPR = Interpersonal Relationship; OVS = Overall Satisfaction. Ceiling (%) represents the proportion of participants selecting the highest response category.

## Data Availability

The original contributions presented in this study are included in the article and Supplementary Materials. Further inquiries can be directed to the corresponding author.

## Supplementary Materials

The following supporting information can be downloaded at: https://www.mdpi.com/article/10.3390/diagnostics16132112/s1, Table S1: Confirmatory factor analysis standardized loadings for the Romanian PSPSQ 2.0 (*N* = 220); File S1: Romanian version of the Patient Satisfaction with Pharmacist Services Questionnaire (PSPSQ 2.0).

## Abbreviations

The following abbreviations are used in this manuscript:

AVE	Average Variance Extracted
CFA	Confirmatory Factor Analysis
CFI	Comparative Fit Index
CITC	Corrected Item–Total Correlation
COSMIN	COnsensus-based Standards for the selection of health Measurement INstruments
DLQI	Dermatology Life Quality Index
DWLS	Diagonally Weighted Least Squares
EBDs	Energy-Based Devices
EFA	Exploratory Factor Analysis
HTMT	Heterotrait–Monotrait Ratio
IPR	Interpersonal Relationship
ISPOR	International Society for Pharmacoeconomics and Outcomes Research
MARS-5	Medication Adherence Report Scale-5
OVS	Overall Satisfaction
PHC	Primary Healthcare
PREMs	Patient-Reported Experience Measures
PROs	Patient-Reported Outcomes
PRMs	Patient-Reported Measures
PSPSQ 2.0	Patient Satisfaction with Pharmacist Services Questionnaire 2.0
QoC	Quality of Care
RMSEA	Root Mean Square Error of Approximation
SRMR	Standardized Root Mean Square Residual
STROBE	Strengthening the Reporting of Observational Studies in Epidemiology
TLI	Tucker–Lewis Index
