# Supplementary material for: Patient-Reported Experiences in Chronic Dermatological Conditions: Validation of the Romanian PSPSQ 2.0 Within Contemporary Dermatologic Care Pathways
Source: Diagnostics (Basel). 2026 Jul 6;16(13):2112. doi: 10.3390/diagnostics16132112 (PMC13361945; doi:10.3390/diagnostics16132112)
Supplement: Supplementary file 1 [file diagnostics-16-02112-s001.zip › File S1 Romanian PSPSQ2 Questionnaire.pdf]

File S1. Romanian Version of the Patient Satisfaction with Pharmacist Services Questionnaire (PSPSQ 2.0).

The following version of the PSPSQ 2.0 was administered to participants during the study.

Vă rugăm să evaluați afirmațiile de mai jos bazându-vă pe experiența din ultimele 3 luni.

Scală de răspuns:

1 = Total în dezacord
2 = Nu sunt de acord
3 = De acord
4 = Total de acord

1. Calitatea îngrijirii oferite de farmacist

Afirmație	Total de acord (4)	De acord (3)	Nu sunt de acord (2)	Total în dezacord (1)
1. Farmacistul a abordat complet problemele mele de sănătate sau îngrijorările mele în timpul vizitei.	☐	☐	☐	☐
2. Farmacistul a fost profesionist în toate interacțiunile noastre.	☐	☐	☐	☐
3. Farmacistul mi-a explicat informațiile clar și ușor de înțeles.	☐	☐	☐	☐
4. Farmacistul s-a asigurat că am înțeles toate informațiile oferite.	☐	☐	☐	☐
5. Farmacistul mi-a oferit suficient timp pentru întrebări și clarificări.	☐	☐	☐	☐
6. Farmacistul m-a ajutat să înțeleg importanța respectării tratamentului.	☐	☐	☐	☐
7. Farmacistul mi-a oferit sfaturi utile despre cum să folosesc medicamentele corect.	☐	☐	☐	☐
8. Farmacistul mi-a oferit recomandări pentru a-mi îmbunătăți sănătatea (ex. dietă, exerciții fizice).	☐	☐	☐	☐
9. Farmacistul m-a ajutat să rezolv probleme legate de tratament (ex. costuri, efecte adverse).	☐	☐	☐	☐
10. Farmacistul a verificat progresul meu în tratament la momentul potrivit.	☐	☐	☐	☐

2. Interacțiunea cu farmacistul

Afirmație	Total de acord (4)	De acord (3)	Nu sunt de acord (2)	Total în dezacord (1)
11. Farmacistul a fost grijuliu și atent la problemele mele de sănătate.	☐	☐	☐	☐
12. Farmacistul m-a încurajat să respect tratamentul pentru a-mi îmbunătăți sănătatea.	☐	☐	☐	☐
13. M-am simțit confortabil în interacțiunile cu farmacistul.	☐	☐	☐	☐
14. Farmacistul m-a tratat cu respect.	☐	☐	☐	☐
15. Farmacistul a fost preocupat de îmbunătățirea sănătății mele.	☐	☐	☐	☐
16. Am avut încredere în informațiile primite de la farmacist.	☐	☐	☐	☐

3. Evaluare generală

Afirmație	Total de acord (4)	De acord (3)	Nu sunt de acord (2)	Total în dezacord (1)
17. Sunt mulțumit/ă de îngrijirea generală oferită de farmacist.	☐	☐	☐	☐
18. Aș recomanda acest farmacist altor persoane.	☐	☐	☐	☐
19. Aș continua să merg la acest farmacist dacă ar fi nevoie.	☐	☐	☐	☐

4. Îngrijirea generală oferită de farmacist

Opțiune	Scor
A depășit așteptările mele	4
A fost așa cum mă așteptam	3
Nu a fost așa cum mă așteptam	2
Nu am avut așteptări	1
