# Supplementary material for: Patient-Reported Experiences in Chronic Dermatological Conditions: Validation of the Romanian PSPSQ 2.0 Within Contemporary Dermatologic Care Pathways
Source: Diagnostics (Basel). 2026 Jul 6;16(13):2112. doi: 10.3390/diagnostics16132112 (PMC13361945; doi:10.3390/diagnostics16132112)
Supplement: Supplementary file 1 [file diagnostics-16-02112-s001.zip › Table S1 CFA standardized loadings for the three-factor model;.pdf]

Table S1. Confirmatory factor analysis standardized loadings for the Romanian PSPSQ 2.0 (N = 220).

Domain	Item	Domain	Standardized loading
QoC	Q1	QoC	0.956
QoC	Q2	QoC	0.971
QoC	Q3	QoC	0.987
QoC	Q4	QoC	0.977
QoC	Q5	QoC	0.915
QoC	Q6	QoC	0.980
QoC	Q7	QoC	0.987
QoC	Q8	QoC	0.916
QoC	Q9	QoC	0.947
QoC	Q10	QoC	0.892
IPR	Q11	IPR	1.002
IPR	Q12	IPR	0.967
IPR	Q13	IPR	0.953
IPR	Q14	IPR	0.974
IPR	Q15	IPR	0.945
IPR	Q16	IPR	1.012
OVS	Q17	OVS	0.998
OVS	Q18	OVS	0.993
OVS	Q19	OVS	0.994

Note: Standardized factor loadings (Std.all) are reported. Values slightly above 1.0 reflect estimation issues (Heywood cases), consistent with high inter-item correlations and model complexity. QoC = Quality of Care; IPR = Interpersonal Relationship; OVS = Overall Satisfaction.
